# A Stated Preference Investigation into the Chinese Demand for Farmed vs. Wild Bear Bile

**DOI:** 10.1371/journal.pone.0021243

**Published:** 2011-07-20

**Authors:** Adam J. Dutton, Cameron Hepburn, David W. Macdonald

**Affiliations:** 1 Wildlife Conservation Research Unit, Department of Zoology, The Recanati-Kaplan Centre, University of Oxford, Oxford, United Kingdom; 2 Smith School of Enterprise and the Environment and James Martin Institute, Said Business School, University of Oxford, and New College Oxford, Oxford, United Kingdom; University of York, United Kingdom

## Abstract

Farming of animals and plants has recently been considered not merely as a more efficient and plentiful supply of their products but also as a means of protecting wild populations from that trade. Amongst these nascent farming products might be listed bear bile. Bear bile has been exploited by traditional Chinese medicinalists for millennia. Since the 1980s consumers have had the options of: illegal wild gall bladders, bile extracted from caged live bears or the acid synthesised chemically. Despite these alternatives bears continue to be harvested from the wild. In this paper we use stated preference techniques using a random sample of the Chinese population to estimate demand functions for wild bear bile with and without competition from farmed bear bile. We find a willingness to pay considerably more for wild bear bile than farmed. Wild bear bile has low own price elasticity and cross price elasticity with farmed bear bile. The ability of farmed bear bile to reduce demand for wild bear bile is at best limited and, at prevailing prices, may be close to zero or have the opposite effect. The demand functions estimated suggest that the own price elasticity of wild bear bile is lower when competing with farmed bear bile than when it is the only option available. This means that the incumbent product may actually sell more items at a higher price when competing than when alone in the market. This finding may be of broader interest to behavioural economists as we argue that one explanation may be that as product choice increases price has less impact on decision making. For the wildlife farming debate this indicates that at some prices the introduction of farmed competition might increase the demand for the wild product.

## Introduction

Damaging illegal trades in the products of the natural world are often tackled by one of two opposing solutions: a total ban on trade or a controlled trade harvested from the wild. However neither the banning of the trade in tiger (*Panthera tigris*) parts [1] nor the controlled trades in fish stocks have halted poaching or the decline in wild populations [2]. The lack of a ‘silver bullet’ to halt illegal wildlife trade leaves scope for dispute between proponents for either of these imperfect cures. Bans are relatively blunt instruments which can be costly and often remove economic incentives to tolerate animals in the wild. Controlled trades from the wild are, conversely, complicated. In some instances a third option is available: to farm wildlife. Wildlife farming offers, at first glance, an intuitively satisfying solution: a legal trade can in principle be created by farming animals to assuage demand for wild animals which thus need not be harvested.

Optimism about farming as a conservation policy is further bolstered by the successes of crocodilian farming in reducing the poaching of wild crocodilians for their skins [3]. However the success of a policy to “farm for conservation” is not certain, and a number of obstacles to its success require consideration. By analogy, a legal trade in mobile phones does not preclude their theft, and crocodilian farms have not entirely removed illegal exploitation [4]. Dutton, Hepburn & Macdonald [5] lists the issues which must be overcome for a farming policy successfully to remove pressure from on the species in the wild, amongst which was substitutability. Here, an illegal trade in wild bear bile and a legal trade in siphoned bear bile acid from farms, for use in traditional Chinese medicine (TCM), are examined for substitutability.

### Bear bile as a medicine

Bear bile is described in the earliest official pharmacopoeia of TCM in AD 659 [6]. TCM practitioners use bile against a variety of illnesses including liver disease, epilepsy and eclampsia [7]. Bear bile would historically have been a scarce and costly product reserved for the wealthy or for serious illness [8]. A wide variety of alternatives are available depending upon the illness. Huang [6] lists 27 alternative species whose bile was said to mimic the effect of bear bile on specific conditions. A WSPA report [9] lists 39 species of flora which might similarly replace bear bile. A non-random survey of 50 TCM practitioners found that 8% felt that bear bile was an irreplaceable and vital part of the pharmacopoeia [10].

Since the availability of bear bile increased due to the production of farms new uses for it have been found, not all of which are supported by TCM. Bear bile shampoos, for instance, might be considered, in western terms, a tonic rather than a medicine. Some TCM practitioners argue that bear bile, being a potent pharmaceutical, is actually dangerous if used as a tonic for regular consumption [8]. There may, therefore, be the emergence of two different trades. The first trade being the traditional use of bear bile as a potent medicine and the second as a tonic: we therefore considered both.

Ursodeoxycholic acid, found in the bile of bears, was first isolated from ursid gall by Shoda et al. [11], and was later produced synthetically by Kanazawa et al. [12]. Today western medicine is using it, or researching its efficacy, against a range of maladies including: liver cirrhosis ([13]), a prophylactic for colon cancer ([14]), to prevent the production of gallstones after surgery [15]. Therefore, the synthetic acid is produced commercially. Ursodeoxycholic acid is produced by all bears, but is found in large concentrations in: polar (*Ursus martimus*), American black (*Ursus americanus*) Asiatic black (*Ursus thibetanus*) and brown (*Ursus arctos*) bears [16].

### Bear welfare and conservation

The bears involved in the bile trade include: American and Asiatic black bears, brown bears, sun bears (*Helarctos malayanus*) and sloth bears (*Melursus ursinus*). However most pressure is placed on the Asiatic black, sloth and brown bear's in Asia ([17]). TCM orthodoxy states that Asiatic black bears and brown bears (incidentally those with the largest levels of ursodeoxycholic acid in their bile) are either most or exclusively desirable ([10]). Gall bladders are relatively small and can be removed from the carcass at the site of the kill for ease of trafficking and the species from which the gallbladder has been taken cannot be distinguished by sight [18]. American black bears are also considered a good source of bile for TCM..

All Asiatic populations of the bear species endemic to Asia are on Text S1 of CITES. Whilst brown bears are of least concern given the overall status of their populations across the globe, East Asian populations remain in Appendix 1. The other bears threatened by the trade in Asia (Asiatic black, sun and sloth) are all considered vulnerable and decreasing by the IUCN [19]. The Asiatic black bears are the most threatened by the trade in gallbladders [20]. Habitat loss is considered the most damaging threat to Asiatic black bears in southern areas such as India, but the trade in bear parts is the major threat to them in China in Southeast Asia [20].

Bears have been farmed for their bile in East Asia since the 1980's when Korean scientists developed a method for extracting bile from live bears through a canula to the bear's bile duct [21]. Over 12,000 bears are estimated to be in farms across China [22], the vast majority of which are Asiatic black bears. A bear can produce 2.2 kg of bile over a 5 year production life [23]. The farming of bears for bile is highly controversial as a result of concerns for the welfare of bears held indefinitely in small cages and enduring either open wounds or regular invasive surgery ([24] [25 {Jeppesen, 2004 #243]). Given the CITES status of the bears, commercial trade between countries in their products is illegal but requires certification.

### Preferences and demand

The TCM practitioner or patient in China could legally choose: farmed bear bile, synthetic bear bile or from an array of alternative species' bile or products from flora. The trade from wild bears continues [17] [26] and so must remain lucrative for some. This paper examines why it might be that farming bears does not preclude poaching, and reveals the magnitude of the effect farming might have on the demand for wild bear bile. We quantify a clear preference for wild caught bear bile over a chemically identical alternative from farms.

Farmed bear bile can be provided in larger quantities at lower cost than the bile of wild bears, and can be supplied through legal channels. As such it is potentially able to compete with a more desirable alternative. In order to measure any conservation effect, in terms of diminished pressure on wild bears, from farming bears we measure the substitutability of the two goods. Substitutability can be estimated by measuring the degree to which the average person would trade wild-sourced bile for farmed-bile at all prices, and then deriving the cross price elasticity of substitution at prevailing prices. Given that the wild trade is illegal adequate market data for these goods do not exist. However, demand can be measured through classic non-market valuation methods such as stated preference techniques [27]. Stated preference experiments ask respondents to imagine a set of options for some policy intervention or product with associated costs and express a preferred option. Respondents in our investigation were asked to imagine they had one of two sets of symptoms and were prescribed bear bile. Given the illness and the prices of the alternative medicines (wild bear bile, farmed bear bile another alternative treatment or nothing) respondents were asked to choose a treatment.

We used stated preferences to estimate demand functions for the two goods at varying prices. In order to measure the impact of farming we gathered data for scenarios where farmed bear bile was and was not available. In order to investigate the use of bile as a tonic, two levels of illness were considered: one serious and the other less so.

The largest single market for bear bile is in the People's Republic of China (henceforth China) given its population and a health care service providing TCM in parallel to western medicine. China has the largest number of bears and bear farms. There is also evidence, from captures and experts, that bear bile is trafficked to China. For these reasons this investigation was focused upon the Chinese population.

## Methods

The University of Oxford's Central University Research Ethics Committee (CUREC) provides a checklist to assess whether research requires ethical audit. Working through this audit revealed that this work would not require a further audit. The checklist was completed and submitted to the IDREC officer.

This study, carried out in the summer of 2008, excluded the semi-autonomous regions and the special administrative regions of China. Sample areas were spread spatially over the length and breadth of the remaining provinces. Table 1 lists the provinces the sample area types and numbers. The survey was carried out following the devastating Sichuan earthquake which lead to some readjustment in sampling from the central areas which is clear from Table 1. Samples were taken in equal numbers from cities, towns and rural areas.

**Table 1:** Sampling points.

city	town	country	Province
86	0	78	Guangdong
90	0	77	Hubei
0	74	0	Shandong
0	76	0	Shanxi
86	74	76	Beijing
88	77	77	Shaanxi
81	77	77	Yunnan
86	74	76	Jiangxi
100	74	73	Shanghai/Zhejiang
90	73	73	Jilin

The official Chinese definition for urban and rural areas places cities and towns in urban areas and villages in rural ones [28]. In 2006, 43.9% of the Chinese population lived in areas designated to be urban whilst 56.1% lived in rural areas. Given the bias built into the sampling procedure, data used in the analysis were weighted to account for this. Percentages reported are adjusted so that rural responses form 43.9% of the total.

Households within these areas were sampled at random using government databases for the area. Individuals within each household were randomly chosen using a KISH matrix [29] but excluded household members which were under 18 or had lived in the sampling area for less than a year. A KISH matrix gathers the name, age and sex of the eligible household members, and then uses a random number to choose which individual to interview.

The survey was administered by Horizon PLC, a China-based professional market research company. Horizon were chosen based upon their previous experience of conservation-related research [30]. Horizon sent representatives to each area to recruit and train locally based interviewers. The interviewees were approached at home, and worked through a questionnaire with the interviewer in return for a small gift of washing powder.

All interviews were carried out face to face by local interviewers in the homes of the interviewees. The data from interviews were returned each day and checked by supervisors; any logical inconsistency or missing/spoiled data at this stage resulted in a re-run. In order to test the returned forms from individual interviewers 30% of interviews were subsequently validated via a phone call by the supervisor and should any have proved to be fraudulent then all questionnaires from the interviewer involved would have been reviewed. Data were further checked at the input stage by the database for syntactical errors.

Initial designs for the questionnaire were prepared in English and pre-tested on 4 Chinese post-graduate students at Lady Margaret Hall, Oxford University, with incremental changes following each to aid understanding of the questions. The survey was then reviewed with the team at Horizon P.L.C. and transformed to aid logical progression through the form and to provide relevant options for multiple-choice questions.

Two rounds of pre-testing, with 10 interviews in each round, were then carried out in Beijing. For optional choices, common responses which had not been considered were added at this stage. Prices of wild bear bile offered in the stated preference section (see below) were adjusted such that some respondents would turn down wild bear bile. In the contingent valuation section, the maximum price of wild bear bile was raised from ¥800 to ¥1500. The primary purpose of the contingent valuation is to estimate demand at likely prevailing prices. A broader spread of prices were used to capture the average maximum willingness to pay for bear bile, resolution would be lost around the prices of interest to the market.

At this stage Horizon produced an English and Chinese version of the questionnaire. The Chinese version was sent to an independent translator to translate it into English. The English translation was then compared against the English version provided by Horizon. No further changes were required as a result of this check on linguistic consistency.

In order to elicit estimates of respondent's preferences and willingness to pay for medicines, the stated preference investigation had three parts. The first encouraged respondents to recall their experiences and knowledge of bear bile; the second elicited their preferences and used debriefing questions to glean insight into their reasoning, and the third gathered demographic information to examine the sampling and for modelling purposes.

Best practice in stated preference investigations requires that sufficient information is provided for the respondent fully to understand the product and the circumstances in which he is asked to state preferences [27]. This is because in most cases the respondent is valuing a public good. In this case, however, the respondent is asked to imagine themselves in a more common purchasing decision of pharmaceuticals rather than a public good. For that reason, rather than giving the respondent more information than they might have in a genuine situation, the first section of the questionnaire asked the respondents' about their experience and knowledge of bear bile. The respondent might then be helped to recollect their own memories and understanding of the product. The first section could then both prepare them to answer stated preference questions regarding wild and farmed bear bile, and directly gather information on experiences.

Respondents were then prepared to enter the choice experiment. Respondents were told that, in the future, there could be a legal trade in bear bile sourced from a sustainable harvest of wild bears. Respondents were told that yields of bile from the wild would be lower, and cost more to manage and obtain, than farmed bile - making wild bile more expensive. For the purposes of the choice experiment, respondents were asked to imagine that the wild bear bile offered came from a legal sustainable supply. In this way the investigation removed the conflating impacts of illegality and subsequent under-reporting due to fear of reprisals. Respondents were asked to state their preferences in four different scenarios. The four scenarios were produced by varying two aspects of the conditions: the health problem faced and the availability of farmed bear bile. Where farmed bear bile was available a choice experiment was used in order to allow respondents to choose: wild bear bile, farmed bear bile, nothing or to seek an alternative. Where farmed bear bile was not available we required information on their propensity to buy wild bear bile and so a simpler contingent valuation approach was used.

The respondent would first be informed of the illness they were to imagine having contracted, before completing a choice experiment and then a contingent valuation exercise.

In the first scenario the respondent was asked to:

“Imagine that you have become very ill. You feel very sick are in a great deal of pain and cannot work. A part of your prescription is bear bile.”

Whilst the second scenario asked them to:

“Imagine you do not have a serious illness but you are uncomfortable. For instance you may get stomach pains, headaches or tired easily. It is strongly suggested by somebody that you trust that you try bear bile.”

A choice experiment typically presents a set of questions where the respondent may choose from a menu of options typically linked to prices. Prices and options vary between questions such that a demand curve may be estimated. In each choice experiment the respondent could choose either farmed bear bile, wild bear bile, seek the closest TCM alternative or to buy none of the above. The prices of farmed bear bile and the alternative treatment were considered to be identical, to accept no treatment was free while a different price was offered for wild bear bile.

The only choice variables involved in the decision were the treatment and its price. As such it is by default a labelled choice experiment. Labelled choice experiments have been shown to distract respondents from the attributes of the choices [31]. However in this case there are no attributes presented and only the price and the respondent's knowledge and biases guide the decision. Moreover labelled choice experiments have been shown to be appropriate in health care economics [32].

Three prices were to be tested for each treatment: high (wild ¥800, farmed ¥56 ), mid (wild ¥300, farmed ¥28) and low (wild ¥80, farmed ¥14 ). These produce 9 possible permutations of prices between the farmed/alternative and wild goods. All respondents were offered the choices with both prices high, both prices low and both prices at the mid-point. In order to test (and adjust) for anchoring [33] the respondents were randomly presented with either the high or the low prices first. Anchoring occurs where respondents use the first price offered to compare prices and so higher prices tend to increase willingness to pay and vice versa.

Depending upon the answers to these three questions many, if not all, of the remaining permutations may be inferred assuming transitivity of preferences. A fourth price combination was then offered dependent upon the answers to the first three to ensure that choices were broadly transitively logical or to allow the remaining permutations to be filled in. As such 9 binary choices could be gathered as a full factorial design for a single respondent with 4 questions.

Following the choice experiments, respondents were debriefed to gain some insight into the reasoning behind their choices. Respondents were asked, by multiple-choice question (with an “other” option), why they had chosen as they had.

Respondents were then asked to consider the same health scenario without the option of treatment with farmed bear bile. Here they were presented with a double bounded dichotomous choice contingent valuation for bear bile alone. Four prices were offered: ¥1500, ¥800 , ¥400, ¥80. Half of all respondents were first asked if they would pay ¥800 and the other half first asked if they would pay ¥400. If they stated that they would pay that price then the higher price was offered and if not then the lower price. The contingent valuation would then end and a debriefing question was asked.

The third section gathered standard demographic information from respondents. This included: age, sex, household income, educational attainment, birthplace and employment status.

The stated preferences were then used to estimate demand functions relating the price of wild and farmed bear bile to consumption of wild bear bile. The quantity of bear bile consumed in the function derived was in terms of the percentage of the population that would buy wild bear bile in the circumstances described with the prices offered. Quantity might more typically be described by volume but we are not here predicting the total number of patients likely to be prescribed bear bile. As such we do not know the volume of bear bile that would be sold and so cannot present these data. Instead we present percentages which might be used by researchers with data on the prescription of bear bile to estimate volumes sold.

The demand functions were then interpreted to aid the debate over whether farmed bear bile might help to reduce consumption of wild bear bile. In order to describe likely impacts of farmed bear bile we chose a number of prices from the literature to enter into the functions. We then calculated the wild bear bile price at which the quantity demanding wild bear bile remains the same when farmed bear bile is and is not available. We also produced cross price elasticities for wild bear bile against farmed bear bile at these points. We finally present the largest probable drop in demand for wild bear bile predicted by the availability of farmed bear bile using a low but possible price for wild bear bile. These steps are described below.

Respondents to the choice experiments could immediately be placed into one of three groups: those who would have bought wild bile at all prices, those who would have bought wild bile at some prices but not others and those who would never have bought wild bile. Insensitivity to price in the first and last groups could have been due to all prices being too high or too low for sensitivity to price to show up in their answers. However some respondents may have considered the products to be non-substitutable and the questionnaire does not differentiate between these motives despite the debriefing efforts. In order to calculate price sensitivity at prevailing prices we used only those who were shown to be sensitive to price to produce demand functions. In estimating the total number willing to pay at a given price, the number from the predicted portion of the population were then added to the number who would have bought at all prices offered.

Using this subset a binary logistic function was used to regress preference for wild bear bile on prices and demographics. The best set of demographic variables to use in the model was chosen based on AIC scores. An alternative model was also produced using a linear regression relating the percentage choosing wild bear bile to the prices of the goods offered only. The contingent valuation results were converted into a demand function using a survivor function [27]. This involves calculating the total percentage of respondents indicating a willingness to buy at each price. Respondents agreeing to purchase bile at a given price are assumed to be willing to buy at all lower prices.

The most useful data come from a 2006 WSPA survey of Chinese pharmacies [34]. All bear bile came as a medical product rather than as raw bile and volumes were not constant. The minimum price for a product was ¥10.1, the maximum ¥594 and the mean ¥93.26 (s.e. 22.05). Few prices for raw bile from farms were found. In 1990 a newsletter reported that it ranged from ¥25.60 to ¥40.00 per gram [23].

We only used wild bear bile where whole gall bladders were for sale and some farmed bile prices were by the gram, with the consequence that a dosage was required to estimate a treatment price. The literature presents a variety of doses for treatment: Huang [6] suggests 0.3–0.6 grams, Lee [8] prescribes 2 grams whilst Mainka and Mills [23] found references indicating doses of between 4 and 11 grams. The literature does not suggest the number of treatments required. We chose a conservative estimate of the amount of bear bile required for treatment at 2.5 grams.

Based on this dose, prices for pure farmed bear bile would then be ¥64.00 to ¥100.00 per treatment. Similar prices have been reported [35] [36] for farmed bile per gram. ¥594 was a single price far in excess of the remaining prices and came from Guangzhou for bear bile capsules [34]. The prices chosen were ¥30 at the lowest end of the mean estimates of prices at 95% significance and ¥90 which is close to the true mean.

Prices for wild bile are poorly documented and variable given the illegality, timescale, geography and lack of formal markets from which these prices are taken. In 1991 Mills [37] found wild gall bladders at a market in Chengdu relatively cheaply for $9–$12/kg, but also found a vendor who would charge $1400–$2700 to bring in a live bear and slaughter it to prove provenance. A gall bladder weighs on average 47–52 grams dried [23]. Wyler [38] estimates for a wild bear gall bladders lead to treatment prices ranging from ¥100 to ¥3,399.

## Results

The response rate was 40.09% (from respondents present and eligible at the time of the interviewer calling) with a total sample size of 1677. The sample is biased towards better educated and wealthier respondents than the overall Chinese population (Table 2 and Table 3). Adjusting for the urban/rural bias still leaves a sample with a greater average education than the populace at large (Table 2).

**Table 2:** Comparing education levels in the sample and in the Chinese Population.

Highest level of Education	Sample	Chinese Statistical Yearbook
None	0.92%	8.79%
Primary School	23.19%	33.07%
Junior School	37.79%	38.99%
Senior School	25.61%	12.93%
College and higher	12.49%	6.22%

The sample values are adjusted for the bias towards urban respondents. Chinese statistical yearbook values are from the 2006 edition.

**Table 3:** Comparing average yearly household income by area type between the sample and the Chinese population.

	sample	china
**URBAN**	43862.44	12719.19
**RURAL**	19724.53222	3587.04

Income and education are correlated. A binary logistic regression of higher education on household income produces a positive coefficient (2.256×10−5 P-value<0.0001). The sex ratio is slightly biased towards women at ∼51% and in the Chinese statistical yearbook 48% of the population are female [28].

33.69% mentioned bile as a part of the bear used in medicine without a prompt. 31.15% of the remaining population then stated that they were aware that bear bile is used in TCM when asked. In total 54.26% of the total sample claimed to have any knowledge of bear bile. The total number who claim to have consumed or known anyone who has consumed bear bile from any source was 19.86% of the sample.

### Bile used for serious illness ∼ scenario 1

Respondents who did not vary their responses showed no sensitivity to price and were therefore excluded from further analysis. Under the first “serious” scenario, correcting for the rural bias, 37.12% were insensitive to price and would buy only wild bile, 38.49% were insensitive to price and bought no wild bile. This left 24.40%, of which 47% were rural, who were sensitive to price. A binary logistic model was then used to estimate the proportion of this 24.4% who might buy legal wild bear bile at prevailing prices.

The lowest AIC score was gained for a model including a variety of demographic variables (Table 4). The model included the prices for both products and a dummy variable for whether the question was the first asked and a separate dummy variable indicating that the higher prices were offered first. Respondents were less likely to buy wild bear bile in the first question asked. It also included the respondent's: household income, sex, whether they were in a rural area and their birth province. We will refer to this as the BL model (binary logistic). To estimate a demand curve, averages from the sample were placed into the model to describe the demographic of the population. For contrast, a second simpler model was produced using a log-linear regression of the percentage of respondents choosing wild bear bile at varying prices of wild and farmed bear bile. This model will be referred to as the LL model (log linear) and shown in Table 5. The equations describing these models can be found in Text S1.

**Table 4:** Chosen binary logistic model of wild bear bile choice in the “serious” scenario.

	Coefficient		P value
Intercept	−9.95×10^−1^	(3.220 e-01)	0.002
Price wild	−1.9×10^−3^	(1.722 e-04)	<0.0001
Price farmed	5×10^−2^	(3.294 e-03)	<0.0001
First price offered	−1.23	(1.424 e-01)	<0.0001
High price first	−1.97×10^−1^	(1.09×10^−1^)	0.07
income	1.08×10^−5^	(2.23×10^−6^)	<0.0001
female	−3.21×10^−1^	(1.04×10^−1^)	0.002
rural	8.4×10^−1^	(2.07×10^−1^)	<0.0001
Higher education	−7×10^−1^	(1.35×10^−1^)	<0.0001
Guangdong	−4.48×10^−1^	(2.68×10^−1^)	0.01
Heilongjiang	1.1	(3.3×10^−1^)	0.0008
Hubei	−4.2×10^−2^	(3×10^−1^)	0.9
Jiangxi	1.62	(4×10^−1^)	<0.0001
Jilin	3.81×10^−1^	(3.71×10^−1^)	0.3
Shaanxi	7.92×10^−1^	(2.97×10^−1^)	0.008
Shandong	3.59×10^−1^	(3.66×10^−1^)	0.33
Shanghai	5.9×10^−1^	(2.65×10^−1^)	0.03
Shanxi	5.52×10^−1^	(3.82×10^−1^)	0.15
Yunnan	1.8×10^−1^	(3×10^−1^)	0.55
Zhejiang	−4.97×10^−1^	(3.56×10^−1^)	0.89

AIC: 2401.9 Correct No 0.62% Correct Yes 0.83% Total Correct 0.75%.

**Table 5:** Log Linear regression of wild bile choice percentages against price under the “serious” scenario.

Variable	coeff	s.e.	p
(Intercept)	0.46	0.10	0.4×10^−2^
log (wild price)	−0.06	0.01	0.3×10^−2^
log (farmed price)	0.1	0.02	0.3×10^−2^

R^2^ 0.8792; P 0.0018.

Table 6 summarises the results of the contingent valuation. The demand function derived is in Table 7.

**Table 6:** Summary of contingent valuation results for the “serious” scenario.

Price (¥)	Rural	Urban	Adjusted total
1500	24.20 (0.45)	30.29 (0.52)	26.87 (0.9)
800	36.88 (0.57)	40.94 (0.59)	38.66 (0.9)
400	51.17 (0.62)	52.32 (0.63)	51.68 (0.8)
100	73.79 (0.47)	65.23 (0.55)	70.03 (0.6)

The number of respondents willing to buy wild bear bile at each price are presented for rural, urban and as a weighted average for all areas as percentages. Standard errors are presented in brackets.

**Table 7:** Log Linear regression of contingent valuation results under the “serious” scenario.

	coefficients	p-value
Intercept	144.26 (6.75)	0.002
Ln(price)	−15.85 (1.08)	0.005

R^2^ 0.995; P 0.005.

**Figure 1:**
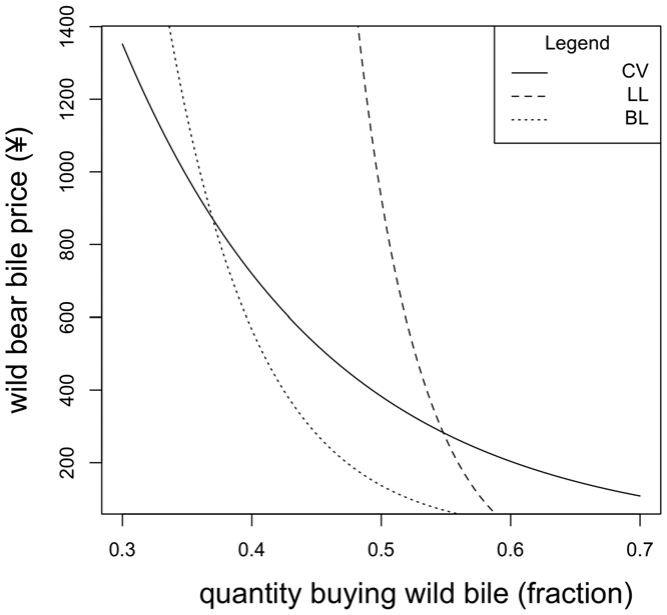
A set of estimated demand functions for wild bear bile. For each function farmed bear bile is held at ¥30 per treatment and these are the results under the “serious” scenario. The CV demand function presents demand in the absence of farmed bear bile whilst the others describe two possible functions when competing with farmed bear bile.

**Figure 2:**
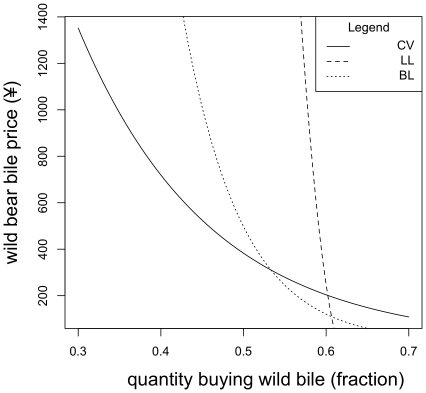
A set of estimated demand functions for wild bear bile. For each function farmed bear bile is held at ¥90 per treatment and these are the results under the “serious” scenario. The CV demand function presents demand in the absence of farmed bear bile whilst the others describe two possible functions when competing with farmed bear bile.

Here we define the prices at which introducing farmed bear bile to the market would be predicted to have no effect in the scenario presented. Setting the farmed price at ¥30 (the lower end of the 95% confidence interval for the average price) the BL model is equal (in total demand) to the Contingent Valuation (CV) model at ¥283.2 for wild bile. That is to say that at this price the BL model predicts no change in demand when farmed bile is and is not available. At this price the cross price elasticity for wild bear bile is 0.13 (calculation of the elasticities is presented in Text S1) indicating that at this point a change in the price of farmed bear bile would have little impact upon the consumption of wild bile. Elasticity of less than 1 indicated inelasticity. This means that a change in price will have little impact upon demand. The Log Linear (LL) model intersects the CV model at ¥871 (cross price elasticity = 0.18). Setting the farmed price at ¥90 the BL model is equal to the CV model at ¥201.2 (cross price elasticity = 0.03); the LL model intersects the CV model at ¥310.2 (cross price elasticity = 0.16). At starting prices above these values the models predict that the public would demand more wild bile, when offered the choice of farmed bear bile, if the price of wild bile either drops or stays the same (see figures 1 and 2).

During this study the lowest retail price derived for a wild bear bile treatment was ¥100 from [38]. It should be noted that other prices are well in excess of this. The effects on quantities demanded at this price are summarised in Table 8. Table 8 describes the predicted reduction in the percentage of respondents choosing wild bear bile between the scenario where farmed bear bile is not, and is, available. It also gives the cross price elasticity of substitution at this price when farmed bear bile is available. Finally it presents the price of bear bile, without farmed competition, from which the drop in price to ¥100, facing farmed competition, would return demand to previous levels. The largest predicted drop in demand was of 19.05% of the population. This drop assumes that the price of wild bear bile does not change. If the price of wild bear bile in the absence of farmed bear bile had been ¥334 or greater, then the demand for wild bear bile would not change or would increase. The negation price reports the price in the single wild bile market which would lead to no change in total demand if farmed bile is introduced and the price for wild bear bile drops to ¥100 per treatment.

**Table 8:** Summary of predicted effects on wild bear bile choice of introducing farmed bear bile maintaining a constant price (100) for wild bear bile under the “serious” scenario.

Farmed price (¥)	Model	Change in wild bear bile consumption	Cross price elasticity	Negation price
30	Lin.	−19.05%	0.16	¥334
	Bin.	−13.49%	0.08	¥235
90	Lin.	−9.96%	0.13	¥310
	Bin.	−10.57%	0.02	¥201

### Bile used for non-serious illness ∼ scenario 2

Under this scenario and correcting for the rural bias 12.41% were insensitive to price and would buy only wild bile, 72.55% were insensitive to price and bought no wild bile. This left 15.04% who chose wild bile at some prices but not others.

A binary logistic model was created for this scenario using the purchase of wild bile as the explained binary variable (Table 9). The lowest AIC score was produced for a model which included no demographic parameters and instead only prices and a dummy variable indicating that the question was for the first price and was the higher option. We will again refer to this in the next section as the BL model. Given that this model included no demographic parameters we did not make a second simpler log linear model for the “non-serious” scenario.

**Table 9:** Chosen binary logistic model of wild bear bile choice in the “non-serious” scenario.

	Coefficient		P value
(Intercept)	−0.52	(0.12)	<0.1×10^−3^
Price Wild	−1.32×10^−3^	(1.67×10^−4^)	<0.1×10^−3^
Price farmed	5.26×10^−2^	(3.42×10^−3^)	<0.1×10^−3^
High price first	−1.57	(0.22)	<0.1×10^−3^

AIC: 2619.8 Correct No 0.57% Correct Yes 0.77% Total Correct 0.69%.

A demand curve was estimated for the contingent valuation using a survivor function adjusted for rural bias (Table 10). The percentage of the population consuming at each price in this survivor function was regressed on the log of wild prices. We will again refer to the log linear regression developed from this survivor function as LL.

**Table 10:** Summary of contingent valuation results for the “non-serious” scenario.

Price (¥)	Rural	Urban	Adjusted total
1500	6.13 (0.56)	15.16 (0.9)	10.10 (0.6)
800	11.36 (0.73)	17.12 (0.90)	13.89 (0.7)
400	21.10 (0.9)	25.74 (0.9)	23.13 (0.9)
100	50.59 (0.9)	46.16 (0.9)	48.65 (0.9)

The number of respondents willing to buy wild bear bile at each price are presented for rural, urban and as a weighted average for all areas as percentages. Standard errors are presented in brackets.

**Figure 3:**
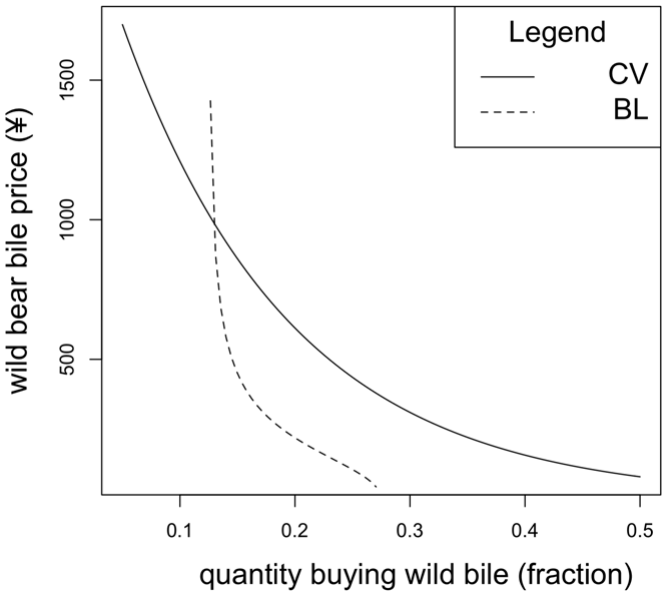
A set of estimated demand functions for wild bear bile. For each function farmed bear bile is held at ¥30 per treatment and these are the results under the “non-serious” scenario. The CV demand function presents demand in the absence of farmed bear bile whilst the other describes demand when competing with farmed bear bile.

**Figure 4:**
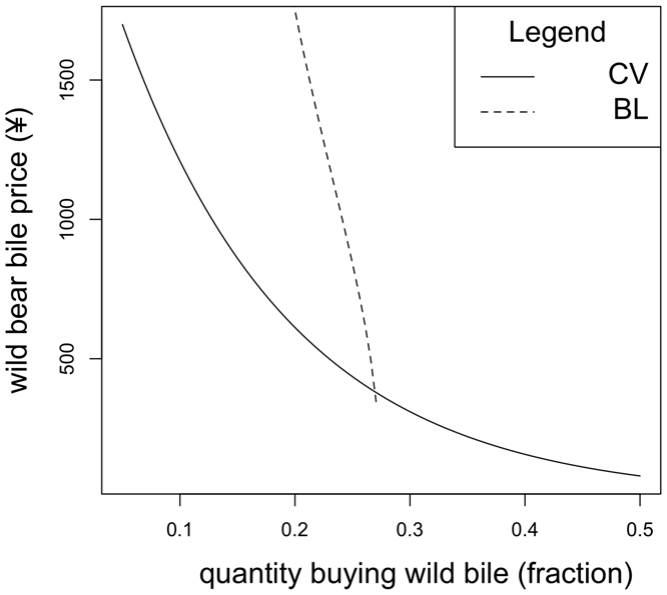
A set of estimated demand functions for wild bear bile. For each function farmed bear bile is held at ¥90 per treatment and these are the results under the “non-serious” scenario. The CV demand function presents demand in the absence of farmed bear bile whilst the other describes demand when competing with farmed bear bile.

Setting the farmed price at ¥30 the demand for wild bear bile, when farmed bile is available, is equal to demand for wild bear bile when farmed bear bile is not offered at ¥569 (cross price elasticity 0.004). Setting the farmed price at ¥90 the demand for wild bear bile when farmed bile is offered is equal to demand for wild bear bile when farmed bear bile is not offered at ¥381.5 (cross price elasticity 0.0002). These functions can be seen in figures 3 and 4.

The effects on the demand for wild bear bile under the second, “less serious” scenario at a wild price of ¥100, both with and without farmed competition, are summarised in Table 11. Table 11 shows the predicted reduction in the percentage of respondents choosing wild bear bile with no change in price as farmed bile is offered, the cross price elasticity of substitution at this price and the price of bear bile without farmed competition which would negate the drop in demand. The largest predicted drop in the percentage of respondents predicted to consume wild bear bile in this scenario was of 23.44%. The preference for wild bear bile persists as when the prices of farmed bear bile and wild are of equivalent magnitude cross price elasticity is low. As the price of farmed bile drops relative to wild bile in the “less serious” scenario cross price elasticity increases. When the illness is “less serious” some consumers are more willing to trade their preferred choice and therefore possibly health for money.

**Table 11:** Summary of predicted effects on wild bear bile choice of introducing farmed bear bile maintaining a constant price (¥100) for wild bear bile under the “less-serious” scenario.

Farmed price	Change in wild bear bile consumption	Cross price elasticity	Negation price
¥30	−23.44%	0.35	¥569
¥90	−19.2%	0.04	¥375

## Discussion

This paper shows that many Chinese people state that they will pay more for wild bear bile than for farmed bear bile. The interactions between the demand functions estimated above present three key results which will further aid in the audit of the efficacy of farming bears for bile as a conservation measure. The first is that the cross price elasticity of wild bear bile with farmed bear bile is inelastic. The second is that, when competing with farmed bear bile, own price elasticity of demand for wild bear bile is relatively inelastic. Finally the demand functions estimated suggest that the gradient of the demand curve, and so the own price elasticity, of wild bear bile is lower when competing with farmed bear bile than when it is the only option available. The preference for wild bear bile, along with the first two findings, indicate that the ability of farmed bear bile to reduce demand for wild bear bile is at best limited and, at prevailing prices, likely to be close to zero. The third finding from the demand functions suggests that at some prices the introduction of farmed competition will actually increase the demand for wild bear bile.

The cross price elasticity of wild and farmed bear bile was at a maximum of 0.2 amongst the price combinations in all models considered, suggesting that substitution from wild bile is inelastic. Expected reductions in demand under optimistic conditions would be less than 20% for serious illness and less than 24% for non-serious. The optimistic conditions were for wild bear bile before and after the introduction of farmed bile to have been at, and remained at, a price much lower than all but one found in the literature. At prices likely to prevail, in the region of ¥1000 per course of treatment according to the most recent reports found e.g. [38], [39], there would be no drop in demand, and no reason to reduce prices given own price inelasticity in the demand for wild bile. Demand functions estimated for this paper indicate that at ¥1000 per wild bile treatment demand for wild bile would increase with the introduction of farmed bile ceteris paribus.

### Price illusion

In order to understand the preferences outlined, we might consider the respondent's understanding of the goods on offer. In this sample a third of respondents are sufficiently familiar with bear bile as a TCM product to refer to it unprompted as a pharmaceutical product of bears. In total, approximately 55% of the sample stated that they were aware that bear bile is used in Traditional Chinese Medicine. Despite a paucity of knowledge, our findings suggest that 54% of the population would buy this good at the prices offered if it were recommended to them for a serious illness. Knowledge of bear bile was not found to be a strong predictor of wild bile consumption and so respondents often chose this expensive alternative purely based on the facts presented in the questionnaire. The only facts given were the prices, the origin of the goods and the prescription, which made no indication as to relative benefits. Notably, whilst being debriefed, of those that chose wild bear bile in the two choice experiments 30.51% and 33.26% respectively stated that it was because they trusted more expensive medicines.

Under these circumstances it is possible that respondents were willing to trust price signals assuming that price would relate to quality. Price will not always provide a clear signal for quality and to some degree the assumption that it does represents an irrational choice similar to ‘money illusion’ [40]. Nearly half of respondents consuming wild bear bile stated plainly that they “trust more expensive medicines”.

The simple assumption that greater prices equate to greater quality is not so dissimilar to the assumption that greater sums of money necessarily lead to greater purchasing power. In circumstances where the consumer is likely to be able to increase the amount of information they have about the good they may be able to rectify this problem. However a sick person will in most cases get better regardless of the treatment and so continue to prefer the more expensive good. Tanaka [41] show how treatments in folk medicine might persist despite a complete lack of any actual medicinal affect.

The majority of consumers did not state that they were led by price. This suggests that the influence of price was either a subconscious one or else they were persuaded by the wild origin of the bile.

Consideration must also be given to the advice given by TCM practitioners who would influence preferences. If practitioners refused to prescribe or offer wild bear bile then consumption would reduce. In such case the key to protecting wild species would not lie with the control of economic markets to alter consumption, but with the TCM practicing community - many of whom agree that there are suitable alternatives to bear bile.

### Lowering own price elasticity as choice complexity increases

We have yet, though, to understand why some respondents appeared to be more likely to choose wild bear bile when farmed bear bile is available than when it is not. Surveys are imperfect reflections of reality. However “People's imperfect knowledge of economic opportunities, their imprudence and unworldliness, have never prevented economists from accepting as basic data the amounts people freely choose at given prices.” [42]. It might therefore be sensible to explore how own price elasticity might reduce for a product when facing new competition.

One explanation may be that as the complexity of the choice increases the price's marginal importance in decision making may wane. Comparing the qualities of one chocolate bar against its price is one level of complexity. Comparing the relative merits of a selection of chocolate bars and their prices reduces price to just one of many considerations and also perhaps “crowds out” the option of having none. As such, providing a choice may, under some circumstances, increase demand for an existing product.

It might be useful to illustrate further what we mean here. The impact of marginal changes in individual product attributes is marginally reduced as the number of attributes or products increases given a cognitive budget. The “resolution” of variable estimates and impacts might diminish as the number of variables increase in a similar way to a person tasked with measuring a wood quickly from a single vantage point. If given the task of measuring the height of the wood, a sample of trees might be measured at close proximity with high accuracy. If two dimensions are required, the width and height of a wood, then a vantage might be chosen further from the subject reducing the accuracy with which the height might be estimated but allowing width and height to be estimated from the same vantage. The argument does not suggest that price is removed from the decision but that small changes in price are more pressing when price is the only variable than when it is one of many to be contemplated.

### Validity

Stated preference studies face a variety of challenges in attempting to ensure that responses reflect the decisions the public would make in real world situations. In order to deal with these challenges a range of tests, best practices and a description of the forms of validity which results must conform to have been produced [27]. Validity testing can be separated into construct and content validity.

Many stated preference studies value public goods in a way in which respondents may not be familiar. Very few people would think about how much they would hope to spend on the defence of realm. A poorly contented valuation instrument would be likely therefore to present a question which the respondent may not understand or may be un-able or unhappy to answer. Such issues are filed under, “content validity”. Given that this stated preference investigation deals with a private good content validity becomes less problematic.

However in order to ensure content validity the interviews went through a process of peer review and pre-testing. The question was framed as one might expect it to be if one were unwell and offered a choice of treatments from a practitioner. The question would not be alien to the respondents. Most notably no respondents refused to answer any of the valuation questions and all respondents were able to understand and give answers to valuation questions.

Construct validity requires that the answers are logically coherent and conform to the predictions of neo-classical economic theory. The preferences of the respondents were shown to be transitive in their preferences 88% of the time. The models from the choice experiments showed that the price coefficients correlated appropriately with price with an increase in the price of the alternative increasing demand (though only very slightly) and a decrease in the price of the good increasing demand. There is also a negative relationship between price and demand for the contingent valuation investigations. Based on these tests the valuation tools can be considered valid.

### Sample bias

There was a bias in the sample towards better off and better educated respondents than might be found in a purely random sample of China. The results suggest that attending higher education establishments made respondents less likely to buy wild bear bile. As such it would seem likely that this bias has reduced the total number willing to buy wild bear bile rather than exaggerated it.

A lower income reduces ability to consume wild bear bile and so would also reduce consumption. Altering the sample population's ability to pay should lower the numbers consuming wild bear bile in both scenarios. As such a reduction in income would not be expected to alter relative results the main finding of this paper would remain intact.

### Limits

There are clear limits to what we can interpret from these results. The results presented here do not represent estimates of the total consumption of bear bile in China. Estimates of total wild bear bile consumption would have to deal with availability, illegality of wild bile, prevalence of relevant diseases and the prescription of medical practitioners. What are clearly represented here are the stated preferences of the consumers.

Without a full understanding of wild bear bile supply it is not yet possible to estimate accurately the reaction of the market to the introduction of farmed bear bile. We can however do some small calculations which indicate that these levels of demand might present a severe threat to wild bear populations at prices presented in this paper. A single bear in a farm might produce an average of 0.44 kg of bile each year [23] and we believe there to be roughly 12000 bears in farms currently [22]. Total supply of farmed bear bile might therefore be of the order of 5.3 tonnes per year. If wild bile were legal our demand functions suggest that at current prices wild bear bile might take up as much as 54% of the market or as little as 12%. Total wild bear demand would therefore be a minimum of 1.38 tonnes per year requiring 27,600 bears. Estimates of the total Chinese population of Asiatic Black bears are between 15,000 and 46,000 [20].

### Conclusion

The results of this manuscript indicate that if the conservation benefits of farming bears are unlikely to be delivered if they rely upon altering the consumption decisions of the final user. The contention of this paper would therefore be that if poaching of bears has been curbed it is most likely because of the illegality of the wild trade in their bile and anti-poaching efforts.

This research concerns the choice of the final consumers, however the medical practitioner may have some influence on this decision which is not captured here. If demand for bear bile is in part driven by the medical professions then there would remain a possibility that farming might have some impact on wild poaching. However there is evidence to suggest that the same preference for wild bear bile can be found within the TCM profession [10] which might undermine this possibility. A further research step might be to interview a large sample of TCM practitioners to gauge how they might react to the loss of a legal supply of bear bile.

Market-based policies such as farming are, however, most persuasively championed when the trade involves disparate and unmanageable groups. By this we mean that if there is a demand for a damaging substance or activity within the populace it might be difficult to curb that demand or prevent supply from illegal sources. If on the other hand demand is largely driven by licensed professionals then it ought to be possible for professional regulatory mechanisms to control their activities thus undermining the argument to facilitate their demand. We are not here suggesting that trade in wild bear bile is being encouraged by the TCM profession, merely acknowledging that we cannot rule this out. As such we might refute this potential argument for bear bile farming were TCM professionals catalysing demand.

The results of this research do not rule out the theoretical possibility that the introduction of farmed bear bile might reduce demand for wild bear bile. However our analysis suggests that any reduction in wild bear bile demand would be partial at best. Moreover under what we posit are the more probable circumstances (namely the higher price estimates) the introduction of farmed bear bile has either had little impact on demand for wild bear bile or in some circumstances increased it.

## Supporting Information

Text S1 (DOC)
