# Supplementary material for: A Stated Preference Investigation into the Chinese Demand for Farmed vs. Wild Bear Bile
Source: PLoS One. 2011 Jul 20;6(7):e21243. doi: 10.1371/journal.pone.0021243 (PMC3140486; doi:10.1371/journal.pone.0021243)
Supplement: Text S1 — (DOC) [file pone.0021243.s001.doc]

**Text S1**

The binary logistic regression predicts the probability that a respondent will consume bear bile based upon the prices of the two products and a range of demographics as shown in equations 1 and 2. If the demographics used are those for the population then we can consider the probability produced to indicate the proportion of the population predicted to purchase wild bear bile and so the quantity sold.

Equation 1 $q=\frac{1}{1+e^{y*}}$

Equation 2

Where pf is the price of farmed bear bile, pw the price of wild bear bile and α is the intercept and the demographics of the individual or population combined.

In order to calculate the cross price elasticity for wild bile consumption with farmed bile price we need to differentiate the model by the farmed price as in Equation 3.

Equation 3

For the log linear model the model the relationship is simpler and shown in equation 4 which and is differentiated in equation 5.

Equation 4

Equation 5

Using equation 3 or 5 we can therefore estimate the cross price elasticity at specific prices for wild and farmed bear bile as in equation 6.

Equation 6
